# Acute cerebellar ataxia as the first manifestation of Imerslund-Gräsbeck syndrome

**DOI:** 10.22037/ijcn.v15i4.27482

**Published:** 2021

**Authors:** Hosein ESLAMIYEH

**Affiliations:** 1.Pediatric Neurology, Department of Pediatrics, Shahid Sadoughi University of Medical Sciences, Yazd, Iran

**Keywords:** Cerebellar ataxia, Megaloblastic anemia, Urinary incontinence, Vitamin B12 deficiency

## Abstract

Imerslund-Gräsbeck syndrome is a rare condition caused by vitamin B12 deficiency and proteinuria. In this article, we reported the case of a 10-year-old girl with imbalance and urinary incontinence. The case had cerebellar ataxia as the primary manifestation. The disequilibrium had progressed gradually within three weeks and was consistent with the symptoms of cerebellar involvement and urinary incontinence. Brain and cervico-thoraco-lumbar magnetic resonance imaging were normal. The patient had elevated lactate dehydrogenase (LDH=4775), in addition to macrocytic anemia, on laboratory examinations; thus, the possibility of malignancy was raised. Then, bone marrow aspiration was performed, showing hypercellular marrow with megaloblastic changes. This finding proved megaloblastic anemia. Regarding the low prevalence of vitamin B12 deficiency in healthy individuals, extensive studies were performed to find out the cause. The serum level of vitamin B12 was found to be lower than the normal range. Although urinalysis revealed significant proteinuria, further nephrological investigations did not indicate any abnormalities. No evidence of serious problems was observed in the gastrointestinal tract study, and metabolic studies were normal. Finally, based on the obtained data, Imerslund-Gräsbeck syndrome was recognized. Patient was treated by vitamin B12 injection, leading to improved balance, and in one-month follow-up, she was able to walk independently, and the cerebellar symptoms had greatly disappeared; however, proteinuria persisted.

## Introduction

Imerslund-Gräsbeck syndrome is a rare syndrome caused by low levels of vitamin B12 (vitamin B12 deficiency) (1). The primary feature of this condition is a blood disorder called megaloblastic anemia (2). In this form of anemia, red blood cells are abnormally large, and its count is diminished (3). About half of people with Imerslund-Gräsbeck syndrome also have high protein levels in their urine (proteinuria) (4). Although proteinuria can be an indication of kidney problems, patients with Imerslund-Gräsbeck syndrome appear to have normal kidney function (5). The incidence of this syndrome is 1:200,000 cases (6). Four hundred cases of this condition have been published and reported so far. This syndrome usually occurs in the first two years of life in the form of anemia with proteinuria as well as neurological symptoms. In this article, we reported a new case of Imerslund-Gräsbeck syndrome with acute cerebellar ataxia as the first manifestation. 

## Case Presentation

A 10-year-old girl was referred to our center due to a lack of balance and urinary incontinence from three weeks ago. The patient was the third child of consanguineous parents. Nervous development of the patient was normal before the onset of disease. The disequilibrium had progressed gradually and was consistent with the symptoms of cerebellar involvement and urinary incontinence. Due to prior low-grade fever, the patient had been treated with the suspicion of viral cerebellitis in the previous center and then referred to us owing to the worsening of symptoms.

During the physical examination, the patient was unable to sit and walk independently, and cerebellar tests, including finger to nose and tandem gait, were abnormal, deep tendon reflexes were diminished, and Babinski sign was detected bilaterally

 Brain and cervico-thoraco-lumbar magnetic resonance imaging was performed for further investigation. These tests were normal. Lumbar puncture was also normal (glucose=50 mg/dl, protein= 30 mg/dl white blood cell = 3, and red blood cell = 0). High concentration of lactate dehydrogenase (LDH=4775) and anemia (Hb=8.8 gr/dl, mean corpuscular volume=104 fL, Red blood cell= 2540000, platelet = 163000) were detected in biochemical tests. Thus, the possibility of malignancy was raised. Organomegaly and lymphadenopathy were not seen in abdominal sonography, and hypercellular marrow with megaloblastic changes was observed in bone marrow examination. In addition to these data, elevated mean corpuscular volume (MCV=104 fL) with hyper segmented neutrophil in peripheral blood smear was noted, and the diagnosis of megaloblastic anemia was established. Consequently, vitamin B12 and folate levels were assessed. The serum level of vitamin B12 was found to be 70.41 pg/ml., which was significantly lower than the normal range (160-970 pg/ml). The range of folate was also normal. 

Regarding the low prevalence of vitamin B12 deficiency in healthy persons, extensive studies have been performed to find out the cause. In order to rule out atrophic gastritis, gastric and duodenal endoscopy and biopsy were performed. The data from the stomach and upper gastrointestinal tract were normal. There was no evidence for celiac disease in duodenal biopsy, and serum anti-tissue transglutaminase (TTG) was within the normal range. Gastrointestinal transit evaluation showed no defect in the mucous membranes of jejunum and ileum. Thus, gastrointestinal disorders were rejected. In urinalysis, 2+ proteinuria was observed with no leukocyturia and hematuria. Further nephrological evaluations, including renal sonography and other specific tests, demonstrated proteinuria (random urine protein/creatine ratio was 1) with an unknown etiology. To rule out the metabolic disorders that can interfere with the metabolism of vitamin B12, chromatography of blood amino acids, urine organic acids assessment, and tandem mass spectrometry were carried out, but no abnormality was detected.

 Finally, based on the investigations and hematologic and nephrological findings, the diagnosis of Imerslund-Gräsbeck syndrome was established. The patient was treated with a high dose of vitamin B12 daily injection (1000 microgram intramuscular) for one week, followed by 1000 microgram weekly, leading to improved balance. In one-month follow-up, she was able to walk, her cerebellar symptoms had greatly disappeared, and the patient had no incontinency; however, proteinuria persisted.

## Discussion

Imerslund- Gräsbeck syndrome, which is an autosomal recessive disorder, was first reported by Lin et al. in China (7). Megaloblastic anemia is the most common clinical manifestation of this disorder because of the selective malabsorption of vitamin B12 (8). The cause of the condition is a defect in the receptor of the vitamin B12-intrinsic factor complex of the ileal enterocyte (9). There are different other manifestations of this disease, including failure to thrive, anatomical anomalies in the urinary tract, and neurological damages (2). Proteinuria without signs of kidney disease is also observed in more than 50% of these patients (10, 11). This malabsorption is related to ileal receptors’ malfunction that is proved by classic Schilling's test. There are three typical features for Imerslund-Gräsbeck syndrome, namely macrocytic anemia, decreased serum B12 level, and proteinuria (12, 13). In our case, macrocytic anemia with megaloblastic changes was detected due to decreased B12 level, while no defect in the mucous membranes of jejunum and ileum was observed. Overall, 50-70% of such patients show proteinuria following megaloblastic anemia (10, 11). 

In our case, this symptom was also detected. We also performed metabolic studies, including urine organic acids and blood amino acid chromatography, yielding no further findings. Based on macrocytic anemia, neurological findings, low level of vitamin B12, and proteinuria, the diagnosis of Imerslund-Gräsbeck syndrome was confirmed. At the time of first visit, the patient was not able to walk. This disequilibrium had progressed gradually and was consistent with the symptoms of cerebellar involvement and urinary incontinence; however, imaging studies were normal. These signs in Imerslund-Gräsbeck syndrome are rare, and to date, no cases with these manifestations have been reported (14). Therefore, the novelty of this reported case lies in acute 

cerebellar ataxia and urinary incontinence as the primary clinical manifestations.

## In Conclusion:

Imerslund-Gräsbeck syndrome is a rare disorder which typically presents with megaloblastic anemia and failure to thrive in infancy ,In this article we present a 10 year-old girl with cerebellar atxia who was eventually diagnosed with Imerslund-Gräsbeck syndrome. This case declares the diverse neurological manifestation of this syndrome and it shows that physicians should be aware about Imerslund-Gräsbeck syndrome when diagnosing ataxia.
